# IMPACT OF OBESITY AND SURGICAL SKILLS IN LAPAROSCOPIC TOTALLY EXTRAPERITONEAL HERNIOPLASTY

**DOI:** 10.1590/0102-6720201700030002

**Published:** 2017

**Authors:** Juliana Mika KATO, Leandro Ryuchi IUAMOTO, Fábio Yuji SUGUITA, Felipe Futema ESSU, Alberto MEYER, Wellington ANDRAUS

**Affiliations:** 1 University of São Paulo Medical School; 2 Liver and Gastrointestinal Transplant Division, Department of Gastroenterology, University of São Paulo Medical School; 3 Abdominal Wall Repair Center, Samaritano Hospital, São Paulo, SP, Brazil.

**Keywords:** Hernia, Body mass index, Herniorraphy, Laparoscopic surgery

## Abstract

**Background:**

Laparoscopic totally extraperitoneal (TEP) hernia repair is a technically demanding procedure. Recent studies have identified BMI as an independent factor for technical difficulty in the learning period.

**Aim:**

To analyze the effect of overweight and obesity on the technical difficulties of TEP.

**Method:**

Prospective study on patients who underwent a symptomatic inguinal hernia by means of the TEP technique. Were analyzed gender, BMI, previous surgery, hernia type, operative time and complications. Technical difficulty was defined by operative time, major complications and recurrence. Patients were classified into four groups: 1) underweight, if less than 18,5 kg/m²; 2) normal range if BMI between 18,5 and 24,9 kg/m²; 3) overweight if BMI between 25-29,9 kg/m²; and 4) obese if BMI≥30 kg/m².

**Results:**

The cohort had a total of 190 patients, 185 men and 5 women. BMI values ranged from 16-36 kg/m² (average 26 kg/m²). Average operating time was 55.4 min in bilateral hernia (15-150) and 37.8 min in unilateral (13-150). Time of surgery was statistically correlated with increased BMI in the first 93 patients (p=0.049).

**Conclusion:**

High BMI and prolonged operative time are undoubtedly correlated. However, this relationship may be statistically significant only in the learning period. Although several clinical features can influence surgical time, upon reaching an experienced level, surgeons appear to easily handle the challenges.

## RESUMO

**Racional:**

A hernioplastia laparoscópica totalmente extraperitoneal (TEP) é procedimento tecnicamente exigente. Estudos recentes identificaram o IMC como um fator independente para a dificuldade técnica durante o período de aprendizagem.

**Objetivo:**

Analisar o efeito do sobrepeso e da obesidade sobre as dificuldades técnicas na TEP.

**Método:**

Estudo prospectivo em pacientes submetidos à hernioplastia inguinal sintomática por meio da TEP. Foram analisados gênero, IMC, operação prévia, tipo de hérnia, tempo operatório e presença de complicações. A dificuldade técnica foi definida pelo tempo operatório, complicações maiores e recorrência. Os pacientes foram classificados em quatro grupos: 1) baixo peso, se menor que 18,5 kg/m²; 2) peso normal se IMC entre 18,5 e 24,9 kg/m²; 3) sobrepeso se IMC entre 25 e 29,9 kg/m²; e 4) obesos se IMC≥30 kg/m².

**Resultados:**

A coorte incluiu 190 pacientes, 185 homens e cinco mulheres. Os valores de IMC variaram de 16-36 kg/m² (média de 26 kg/m²). O tempo médio de operação foi de 55,4 min nas hérnias bilaterais (15-150) e 37,8 min nas unilaterais (13-150). O tempo cirúrgico foi estatisticamente correlacionado com o aumento do IMC nos primeiros 93 pacientes (p=0,049).

**Conclusão:**

IMC elevado e tempo operatório prolongado estão indubitavelmente correlacionados. No entanto, esta relação pode ser estatisticamente significativa apenas no período de aprendizagem. Embora várias características clínicas possam influenciar o tempo cirúrgico, ao chegar a um nível experiente, os cirurgiões parecem lidar facilmente com os desafios.

## INTRODUCTION

Obesity and overweight has been considered an increasing global problem over the last three decades. The World Health Organization estimates a total of 700 millions obese and 2.3 billion overweight adults by 2015. Besides directly linked to several deseases including hypertension, diabetes mellitus and cardiovascular diseases^8^ ^,^ ^36^, higher health costs also contributes to the increased political awareness to take action against it ^14^.

Body mass index (BMI), which is calculated by dividing weight in kilograms by height in meters squared, is the most accepted measurement of obesity. BMI lower than 18,5 kg/m² is considered underweight; between 18,5 and 24,99 kg/m² is the normal range; exceeding 30 kg/m² is defined as obese. Several studies have demonstrated the effects of high BMI on surgical procedures, postoperative complications and anesthetics risks^2^ ^,^ ^16^ ^,^ ^24^ ^,^ ^32^. A meta-analysis of Liu et al. revealed high risks of surgical site infections and pulmonary infections after gastrointestinal procedures^17^. Desai et al. described high rates of skin necrosis, hernia recurrence and necessity of reoperation^6^. Takiguchi et al. correlated obesity with high mortality^32^. In terms of inguinal hernia repair, authors already presented an increased risk for postoperative complications and recurrence^3,18, 23,25,28^.

Endoscopic approach is widely accepted technique for hernioplasties among obese^12^. Between laparoscopic totally extraperitoneal (TEP) and transabdominal preperitoneal (TAPP) hernia repair, the first is preferred since it avoids intraperitoneal approach^13, 21^ and provides less postoperative pain and fast recovery^19^. Recent studies have suggested variants of TEP approach using 2-port, minimizing postoperative complications^10^. Few studies have evaluated the influence of high BMI on hernioplasties operative time. Akagi et al. presented a statistically significant correlation between BMI and technical difficulty during laparoscopic anterior resection^1^. However, Park et. al. demonstrated that BMI was a significant factor influencing surgical difficulty only in the learning period^26^. Apart from surgeon’s expertise, several clinical characteristics may influence operative time. 

The aim of the present study was to analyze the effect of overweight and obesity on the technical difficulty of TEP performed by a single experienced surgeon.

## METHODS

After Ethics Committee approval, a prospective study of patients who underwent a symptomatic inguinal hernia by means of the TEP technique between May 2009 and May 2014 was performed. Medical records from patients operated by a single senior surgeon were analyzed in terms of previous surgery, BMI, type of hernia, operative time and complications. Technical difficulty was defined by prolonged operative time and major complications. All patients signed an informed consent form.

Patients were classified into four groups: 1) underweight, if BMI less than 18.5 kg/m²; 2) normal range, if BMI between 18.5 and 24.99 kg/m²; 3) overweight, if BMI between 25 and 29.99 kg/m²; and 4) obese, if BMI≥30 kg/m².

### Statistical analysis

Variables were analyzed by Sperman’s correlation. Statistically significant values were defined as p<0.05^9^ ^,^ ^27^. For statistical purpose, patients were divided into three groups: A - general, B- unilateral surgery, and C - bilateral surgery. Complicated cases were excluded for correlation analysis, since they could produce bias in length of operation. 

## RESULTS

Among a total of 238 patients identified on the period of study, sufficient data was obtained from 190.

There were 185 men (97.4%) and five women (2.6%). Average age was 52 years, average BMI was 26 and average time of surgery was 44 min (Table 1). Types and percentages of hernia are shown in Table 2.

**TABLE 1:** Descriptive analysis of age, body mass index and operative time

	n	Mean value	Median value	Standard deviation	Range	1st quartile	3rd quartile
Age	190	52.31	52.00	14.85	10-85	42.00	62.25
BMI*	190	25.83	25.00	3.34	16-36	23.00	28.00
Operative time	190	44.02	40.00	21.54	13-150	30.00	54.00

* BMI=body mass index

**TABLE 2:** Type of hernia

Type of hernia	n	%
Direct	60	31.6
Indirect	112	58.9
Femoral	3	1.6
Spiegel	1	0.5
Recurrent	29	15.3
Bilateral	80	42.1
Unilateral	110	57.9

Average operative time was 55.4 min per bilateral hernia (15-150) and 37.8 min per unilateral (13-150). 

Among patients with BMI≤24.99 kg/m², 36 had bilateral hernia; 25 at least one previous surgery; and four postoperative complications, including two conversion to open surgery, one haematoma, one cord edema and one hernia recurrence. Average operative time in this group was 41.6 min (16-120). Overweight patients presented 35 bilateral hernias; 32 had previous surgery; and six had complications: three conversions, one haematoma, one hematuria and one seroma. Average operative time was 43.8 min (13-150). Among 22 obese, nine had bilateral hernia; 10 underwent a previous surgery; and no complications were seen. Average time of surgery was 51.9 min (20-130).

**FIGURE 1:**
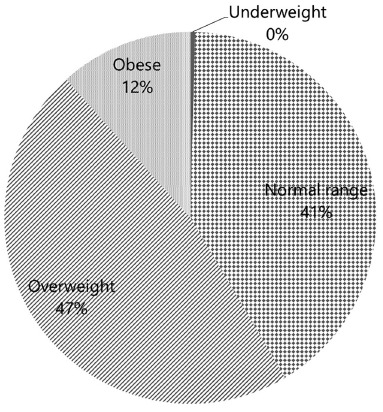
Groups according to patient’s BMI

Distribution according to BMI is shown in Figure 1.

According to ASA classification, 40% were classified as ASA 1, 55.8% as ASA 2 and 4.2% as ASA 3. Four patients (2.1%) stayed longer than 12 h in hospital.

In group A, time of surgery was statistically correlated with increased BMI in the first 93 patients (p=0.049). Among patients who underwent unilateral hernioplasty (group B) p value was 0.42. Best p value was obtained from the first 14 patients (p=0.083). Patients who underwent bilateral surgery (Group C) had the best p-value obtained from the first 42 patients (p<0.001, Table 3).

**TABLE 3:** Correlation between operative time and hernioplasties

	n	p	Best p value	n of first patients for best p value
General (group A)	190	0.14	0.049	93
Unilateral (group B)	103	0.42	0.083	14
Bilateral (group C)	77	0.07	<0.001	42

## DISCUSSION

Among this cohort of 190 patients, BMI values ranged from 16-36 kg/m² (average 25.8 kg/m²) and there was a prevalence of men (97%), similar to other hernia studies ^28^ ^,^ ^29^. Mean time of surgery (44 min) is also in accordance with other authors.

This series demonstrates a significant correlation between BMI and operative time, suggesting that higher BMI is related to more technical difficulty. Not only anatomical factors, but also the higher prevalence of obesity-related comorbidity (hypertension, diabetes, dyslipidemia) can make surgical procedures in obese patients more difficult. Akagi et al. presented a statistically significant correlation between BMI and technical difficulty during laparoscopic anterior resection^1^. It occurs mainly due to: 1) uncontrolled bleeding, since there is a release of angiogenesis-related growth factors by adipose-derived stem cells, which expands the capillary network^7^ ^,^ ^33^; 2) abundant fat tissue, demanding more time to dissect; 3) diagnosis of inguinal hernia in obese can be delayed as the surrounding abdominal fat may hide the problem, therefore it can produce irritation of the hernia sac and lead to more complicated inguinal hernia^35^. In the series of cases operated by TEP, overweight was associated with longer operative time, being statistical significant^30^.

Nevertheless, the correlation was statistically significant only in the first 14 patients who underwent a unilateral hernia (p=0,083) and in the first 42 patients who underwent a bilateral hernia repair (p<0,001). It suggests an influence of a learning curve on technical procedure. Due to unfamiliar pelvic anatomy and limited working space, TEP hernioplasty requires time to achieve excellence. This gap is more affected by patients’ clinical characteristics and anatomical aspects. Previous studies have affirmed that BMI may be a significant factor influencing surgical difficulty only in the learning period^15, 26^. Estimated number of surgeries ranges from 30-60 in literature^4,15, 26^. Arriving at an experienced level, it seems that surgeons can deal with challenges proficiently. 

Comparing bilateral and unilateral hernias, the first obviously demands longer operative time and has worse postoperative complication according to Jacob et. al.^11^. This probably explains the positive correlation between operative time. Studies have demonstrated preference of laparoscopic methods over open techniques, mainly due to the possibility to reach the contralateral side through the same incision^5^ ^,^ ^20^ ^,^ ^22^ ^,^ ^34^. Among laparoscopic approach, the prospective randomized trial of 60 patients of Sharma et. al. showed similar outcomes between TEP and TAPP for bilateral inguinal hernias^31^.

Difficulty associated with BMI in the learning period can help young surgeons to select hernia patients in order to overcome the learning period easily. Since this study included a series of cases of a single surgeon, further studies gathering young surgeons could be helpful to better evaluate the correlation between high BMI and operative time. 

## CONCLUSION

High BMI and longer operative time are undoubtedly related. However, this correlation may be statistical significant only in the learning period. Although several clinical characteristics may influence operative time, when arriving at an experienced level surgeons seem to deal with challenges easily.
